# Caries Experience of Adults with Intellectual Disability in the Western Part of Germany

**DOI:** 10.3390/jcm10122602

**Published:** 2021-06-12

**Authors:** Peter Schmidt, Michael Egermann, Claudia Sauerland, Andreas G. Schulte

**Affiliations:** 1 Department of Special Care Dentistry, Witten/Herdecke University, 58455 Witten, Germany; Michael.egermann@uni-wh.de (M.E.); andreas.schulte@uni-wh.de (A.G.S.); 2 Dental Health Service, Health Authority of Unna District, Platanenallee 16, 59425 Unna, Germany; claudia.sauerland@kreis-unna.de

**Keywords:** caries prevalence, oral health, sociodemographic factors, living arrangements, epidemiology, dental treatment need, fissure sealant, special care dentistry, special needs patients

## Abstract

Background: In Germany, there is limited evidence on the oral health of adults with intellectual disabilities (AwID). Methods: In 2017/18, dental examinations of AwID and a questionnaire survey of their legal guardians were carried out. The mean D3MFT values were calculated to describe the caries experience. The prevalence of AwID with at least one fissure sealant (FS) was determined and associations between caries experience and various sociodemographic factors (e.g., age, gender, living arrangements) were investigated. Results: The data of 132 AwID (mean age 35.2 years; range 18–69 years) could be included. For all AwIDs the mean D3MFT value was 9.5 (95% CI 8.1–11.0). The mean D3MFT value for the 35–44-year-olds was 10.9 (95% CI 8.4–13.4). All caries-free persons (*n* = 14) were younger than 45 years. Furthermore, the mean D3MFT value for AwID living with their parents was lower at a statistically significant level than that of AwID in independent living arrangements. Moreover, younger AwIDs (18–34-year-olds) with at least one FS had a statistically significantly lower mean D3MFT value compared to those without any FS (D3MFT: 3.0 vs. 6.7). Conclusions: The dental health of AwID has improved in Germany in recent years, but, on average, AwIDs still have more missing teeth than their peers in the general population. Oral epidemiological studies on AwID should include information on their living arrangements to assess potential associations between sociodemographic factors and oral health.

## 1. Introduction

Caries ranks amongst the most prevalent chronic diseases. Globally, in 2017, there were 2.3 billion people who had untreated caries in permanent teeth [1]. In fact, between 1990 and 2017, its prevalence in permanent teeth increased by 36%, worldwide [1]. In contrast, in Germany, caries prevalence and caries severity continuously decreased in nearly all age groups [2,3,4]. Between 2005 and 2014, the mean DMFT of 35–44-year-olds decreased from 14.5 to 11.2 [2,4]. In various European countries mean DMFT values ranging between 6.6 and 17.6 were reported for this age group [5]. For young seniors (65–74 years old) from Germany, the mean DMFT was reported to be 17.7 [2]. Although this trend of decreasing caries experience appears to be ongoing, an improvement in oral health cannot be seen in equal extent in all age groups and subgroups of the German population [6]. In some societal subgroups—which mainly include persons with intellectual, physical, and/or sensory disabilities—oral health is still distinctly poorer than in the general population. The finding that the proportion of adults with intellectual disability who are edentulous, have inferior periodontal health, or have only few restored teeth is higher than that of adults in the general population has also been substantiated in international systematic reviews [7,8,9]. Similarly, in Germany, children, adolescents, and adults with intellectual disability have been found more likely to suffer from a higher number of carious or missing teeth than individuals in comparable age groups in the general population [10,11,12,13,14]. Despite these findings, the number of scientific publications from Germany investigating oral health in adults with intellectual disability has been surprisingly small over the last decades [14]. To date, representative national studies on the oral health of persons with disability are lacking in Germany, just as in other countries. In Germany and several other countries, the current state of knowledge about the oral health status of adults with disability is, therefore, based mainly on the results of the few previous studies that were conducted on two different types of sample populations [8,9,15,16]. In these German studies, the investigated sample populations had been either athletes with intellectual disability taking part in the national summer games of the Special Olympics Germany, or individuals occupied in sheltered workshops for persons with intellectual disability [14].

The last published investigation on the oral health of adults with disability in Germany was conducted in the federal states of Sachsen and Baden-Württemberg in 2007 and 2008 [11]. To date, a more recent study investigating oral health in a comparable population of persons with intellectual disability in Germany is, thus, lacking. The main aim of this study was, therefore, to determine caries prevalence and experience in a population of adults with intellectual disability occupied in sheltered workshops, for a region of the federal state of Nordrhein-Westfalen, the most populous German federal state. A secondary aim was to investigate the potential association of sociodemographic factors (gender, age, and living arrangements) and epidemiological caries data for persons with intellectual disability. The hypothesis was that the oral health of persons with intellectual disability living with their parents or other family members would be better than that of those living in assisted living facilities or living independently (i.e., alone, with a partner, or in shared accommodations). A further aim was to analyze whether the availability of different caries preventive measures that were introduced to the German health care system about 30 years ago had positively affected the oral health of the young adults examined in the present investigation. A final aim of the study was to compare the current results with those from the last oral health study in Germany, conducted 10 years previously, on adults with intellectual disability occupied in sheltered workshops.

## 2. Materials and Methods

The present epidemiological cross-sectional study was conducted in cooperation with the Dental Health Service of the Health Authority of the District of Unna, Nordrhein-Westfalen, Germany. The ethics committee of the Witten/Herdecke University gave a positive vote prior to the start of this study (#70/2017).

***Study Region***: The administrative district of Unna is located in the centre of the German federal state of Nordrhein-Westfalen. The district has 395,000 inhabitants and covers 10 municipalities [17]. In 2018, the average income of private households in this district, available for consumption and savings, was 21,494 €, a sum that corresponds closely to that of 21,952 € for Germany as a whole [18,19].

On 31 December 2017, the reported number of persons with disability living in the administrative district of Unna was 50,667. This places the share of persons with severe disability in the district at the time of the study at 12.9%, a figure slightly above the respective figure of 10.2% for the entire federal state of Nordrhein-Westfalen [17]. For Germany, the percentage of people with severe disability was 9.4% at this time [20], while at the EU level, 7.5% of EU citizens aged 16 and more self-reported being affected by severe health-related limitations in their daily activities [21].

***Study Sample***: For the present study, the study participants (i.e., adult persons with intellectual disability occupied in sheltered workshops) received a dental examination between September 2017 and July 2018. These workshops, which are run by the Perthes Foundation charity, are located in the municipalities of Bergkamen, Kamen, and Unna in the district of Unna. There, the dental examinations were also carried out.

Prior to the investigation, the legal guardians of the study participants had been asked to complete a questionnaire composed of 47 questions, based largely on the questionnaire used by Schulte et al. (2013) in 2007 and 2008 [11]. The purpose of the questionnaire was to obtain sociodemographic data and details on the provision of dental care in the investigated population. Legal guardians who felt incapable of completing the questionnaire themselves were asked to consent to completion of the questionnaire by other caregivers involved in the participants’ health care, e.g., other family members or social education workers in assisted living facilities. The dental examinations were only carried out after written consent had been obtained either from the research participants or their legal guardians. In addition, the dental examinations were only carried out if the participants still volunteered to be examined on the day of the examination. Although this approach may seem self-evident, it is of importance to underscore the fact that none of the participants from the sheltered workshops were persuaded, or forced, to accept a dental examination. Additionally, the study participants were given the opportunity to look at an informative video showing the dental examination procedure, e.g., before the actual examination began.

***Examiner and Training***: The examiner who carried out the dental examinations was a trained, very experienced dentist (AGS), who had previously participated in several other oral-epidemiological studies and had trained other dentists for the same task. The only tools used in the examinations were dental mirrors, blunt dental probes, and artificial light (portable halogen lamp). The examinations took place in the sheltered workshops. Missing teeth, dentine carious lesions, restorations, crowns, bridges, dentures, and fissure sealants were documented following the 2013 WHO recommendations [22]. No auxiliary tools for caries diagnostics (i.e., radiographs, FOTI or Laser) were used in this study. The same criteria and procedures that been applied for the dental examinations of persons with intellectual disability occupied in sheltered workshops in the previous surveys for two other German regions in 2007 and 2008 were also used in this study [11]. Further details can be found there.

***Transfer and Assessment of Data, and Statistics***: The findings of the dental examinations were recorded on standardized (paper) sheets. The questionnaires were also in paper-pencil format. To be able to match the dental findings with the responses from the questionnaires, both papers for the same participant were coded with a pseudonym in the Department for Special Care Dentistry of Witten/Herdecke University. The data from these paper records were then entered into the electronic program Microsoft Office Excel, version 2016 (Microsoft Corp., Redmond, Washington, USA), which was also used for the statistical evaluation. The inclusion criteria for this study were informed consent, and the availability of both the dental findings and the completed questionnaire.

The mean D3T-, MT-, FT-, and D3MFT values and their standard deviations were calculated to describe the caries experience. The proportion of adults with D3MFT = 0 was used to calculate the caries prevalence rate. Furthermore, the prevalence of study participants with at least one fissure sealant (FS) was determined. A fissure sealant was regarded as being present even if it only partially covered the occlusal fissure system. Finally, the 95% confidence interval (95% CI) was calculated for all of the variables mentioned above. The resultant values were used to determine whether the difference between two groups was statistically significant.

In addition, associations between caries experience and various sociodemographic factors (e.g., age, gender, and living arrangements) were investigated. The study participants were assigned to three groups on the basis of their living arrangements. Group 1 comprised participants who were living with a family member (e.g., parents). Group 2 comprised participants who were living in assisted living facilities for persons with disability. Group 3 comprised participants who were living independently, i.e., alone, with a partner, or in shared accommodations.

## 3. Results

**Figure 1:**
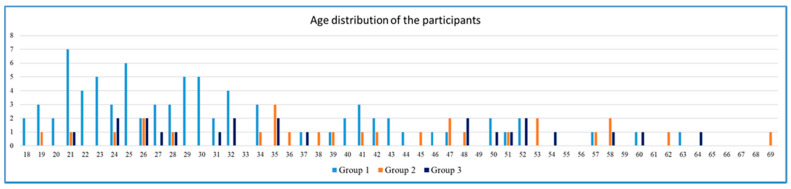
Age distribution of the participants assigned to Group 1, Group 2, or Group 3, according to their living arrangement.

In total, 847 legal guardians of persons with intellectual disability occupied in sheltered workshops were asked for their consent to the dental examination of their wards. In addition, they were requested to complete a prepared questionnaire. Consent for the dental examination was obtained for 137 persons (16.2%). The questionnaires were completed by 161 persons (19.0%). For the present study, the data of 132 (15.6%) persons with intellectual disability, aged between 18 and 69 years, could be included because both the dental record and a completed questionnaire were available. The average age of the included participants was 35.2 years old (SD 12.7). The proportion of men was 49.2%. The majority (61.4%) of the study participants were living with their parents or other family members. Further figures to characterize the study group are presented in Table 1. The mean age of the members of the three groups ranged from 31.2 to 42.0 years (Table 2). In these groups, the age distribution differed distinctly, as Group 1 was dominated by 18–32-year-olds, while Group 3 consisted of persons between the ages of 21 and 37 years and persons between the ages of 48 and 64 years (Figure 1).

***Caries Experience and Caries Prevalence***: The caries prevalence in all study participants together was 89.4% (95% CI 87.6–91.2). The respective proportions for men (87.1%; 95% CI 85.3–88.9) and women (91.0%; 95% CI 89.2–92.8) were nearly the same. All 14 participants (10.6%) without caries experience (D3MFT = 0) were younger than 45 years (Table 2). The proportion of study participants with previous caries experience, but no current need of treatment was 72.7%. This figure was nearly the same in all three groups. The mean D3MFT value for all study participants was 9.5 (95% CI 8.1–11.0) and was nearly the same both for men and women. Furthermore, the observed mean MT values were lower than the mean FT values. Additional details are presented in Table 2 and Table 3.

***Association between Caries Experience, Caries Prevalence and Living Arrangements***: For Group 1, a caries prevalence rate of 91.4% (95% CI 89.6–93.2) was calculated. The respective values for Group 2 were 89.3% (95% CI 87.5–91.1), and 81.8% (95% CI 80.0–83.6) for Group 3. Thus, caries prevalence was, at a statistically significant level, lower in Group 3 than in Groups 1 and 2. In regard to caries experience (mean D3MFT values), this statistically significant difference was only true for comparison of Group 3 with Group 1. In respect to the singular components of the D3MFT score (D3T, MT, FT), the study participants in Group 3, on average, had more missing teeth (MT = 8.6; 95% CI 4.3–13.0) than those in Group 1 (MT = 2.9; 95% CI 1.8–3.9) or in Group 2 (MT = 5.2; 95% CI 2.1–8.4). Again, a statistically significant difference was only true for comparison of Group 3 with Group 1. Attention should, however, explicitly be drawn to the fact that the mean age of the study participants in Group 3 was 40.4 years, while the mean age in Group 1 was 31.2 years. See Table 2 and Table 3, Figure 1 for further detail.

***Association between Mean DMFT Score and Presence of at least One Fissure Sealant in Younger Adults***: Because the proportion of participants with at least one fissure sealant was only 10.3% (*n* = 6) in the age group of 35–69-year-olds, as opposed to 50.0% (*n* = 37) in the young adults in the age group of 18–34-year-olds, only the latter age group was analyzed in detail for an association between DMFT and the presence of fissure sealants. Here, the mean D3MFT value was statistically significantly lower in the study participants with at least one fissure sealant compared to those without any fissure sealant (D3MFT: 3.0 vs. 6.7). More values are given in detail in Table 4.

***Group Aged 35–44 Years Old***: To be able to compare the results of the present study with other regional, national, and international studies, the caries epidemiological data were calculated for the WHO target age group of 35- to 44-year-olds. In the present study, 4.2% of the participants in this age group were caries-free, while the proportion of participants with at least one fissure sealant was 20.8% (Table 2). The mean D3MFT value was 10.9 (95% CI 8.4–13.4). Similarly, to the age group of 18–34-year-olds, the mean MT-value was distinctly lower than the mean FT value in the 35–44-year-olds (Table 3). In our study, 37.5% of the participants in this age group had received prosthodontic treatment (Table 2).

## 4. Discussion

Substantial conclusions about the dental health of adult persons with intellectual disability occupied in sheltered workshops and living in different living arrangements can be drawn from the data compiled in this study. To the authors’ best knowledge, this is only the second study to be published on to investigate dental health in a population of adults with intellectual disability occupied in sheltered workshops in Germany since 2000 [14]. The first study was conducted between 2007 and 2008 on a comparable study population in the German federal states of Baden-Württemberg, located in the south-west of Germany, and Sachsen, located in the south-east [11]. Although the database on the dental health of children and adolescents with intellectual disability is slightly more extensive than that for adults, the number of studies conducted in children and adolescents in this population is also still low [14]. This backdrop illustrates the scantness of published data on the dental health of these patient populations in Germany and underscores the importance of a more current study on a population of adults with intellectual disability in Nordrhein-Westfalen, the most populous federal state in Germany.

A hypothesis in this study was that caries experience as expressed by D3MFT values would differ according to the participants’ different living arrangements. Because the mean D3MFT value for study participants who were living with their parents (Group 1) was lower at a statistically significant level than that of study participants in independent living arrangements, i.e., alone, with a partner, or in shared accommodations (Group 3), the results of the present study appear to bolster this hypothesis. Since the mean age and number of the persons in each of the three groups differ distinctly (Figure 1, Table 2 and Table 3), this finding, however, needs to be regarded critically, and the observation should better only be seen as a tendency. Statistically, the caries experience of study participants who were living in assisted living facilities did not differ significantly from that of the other two groups in this study (Table 3). Although in the previously published study from Germany, conducted in 2007 and 2008, the mean D3MFT index of study participants living with their parents was, statistically, significantly lower than that of study participants living in assisted living facilities, the authors of that study explicitly point out that the mean ages of these two groups differed distinctly [11]. It should also be noted that, in that study, the study sample had only been divided into two groups (persons living with their parents and persons living in assisted living facilities). At that time, no group of individuals living in independent living arrangements (alone, with a partner, or in shared accommodations) could be identified. This fact can perhaps be ascribed to the circumstance that social education workers have only in recent years begun encouraging people with disabilities to live as independently as possible.

Based on the authors’ own clinical experiences, the observation that the dental health of persons with intellectual disability living in supervised environments (parents or assisted living facilities) is better than that of those in independent living arrangements does not seem improbable. Although persons with intellectual disability in independent living arrangements are essentially able to manage everyday life and can also, from a dental point of view, be regarded as capable of brushing their teeth without support, they can lack sufficient insight to brush their teeth daily. In addition, due to the lack of regular supervision in these settings, the efficiency in performing this task is not monitored. In 2005, a dentist with extraordinary experience in providing dental care to persons with disability already noted that even individuals with intellectual disability who are able to brush their teeth alone, or insist on doing so alone, need a certain measure of support, such as regularly being motivated to follow a daily toothbrushing routine and regularly being checked in regard to its proper execution [23].

Basically, these considerations elucidate how extremely important professional dental care, with a focus on prevention, is for the population of persons mentioned above. In our study, in Group 3, the mean number of missing teeth (MT = 8.6) and the proportion of individuals who had received prosthodontic treatment (50%) was distinctly higher than in the two other groups (Table 2). These observations show that many of the individuals in Group 3 had not experienced a sufficient degree of conservative dental therapy or professional preventive dental care. A similar observation is reported in a study from Scotland that compared data on dental care for persons with intellectual disability with such data for the general population [24]. In addition, two international reviews have also shown that a lower number of teeth with restorations are, on average, found in adults with intellectual disability than in adults from the general population [8,9]. Because a high proportion of the participants in Group 3 of our study were either caries-free or were missing many teeth, and therefore had only little need for treatment (Table 2 and Table 3), their dental status, at first glance, appears contradictory to the published findings. This contradiction can, however, be explained by the peculiar age distribution in this group, in which half of the participants were between 18 and 34-years old, and the other half were between 45 and 69 years old (Figure 1). In light of the age distributions in Group 3, with many of the young adults caries-free and many of the older adults missing teeth, the low need for dental treatment is comprehensible.

The results of the present study, which indicate that the dental health of adults with intellectual disability has improved in Germany over the last decade, are pleasant to share. The proportion of persons in this population who do not require dental treatment has thus doubled from 36.2% to 72.9% within this time span [11]. The same positive development could be observed in regard to the proportion of persons who were caries-free as, while in 2007/2008, this proportion was 4.5% [11], it was 10.6% in the present study. Furthermore, the mean D3MFT value decreased from 12.3 (95% CI 11.6–12.9) in 2007/2008 [11] to presently 9.5 (95% CI 8.1–11.0), a difference which proved to be statistically significant. This improvement is mainly due to a decrease in the mean number of missing teeth from 5.7 [11] to 4.3, and partially due to a decrease in the mean number of carious teeth from 2.0 [11] to 0.5 (Table 3). This positive development corresponds to a general improvement in dental health in the general population in Germany over the last three decades. According to the report on the 4th and 5th national German Oral Health Study [2,4], the mean D3MFT score in persons aged 35–44 years has decreased from 14.5 to 11.2 during this period and the mean number of missing teeth (MT) has dropped from 2.4 to 2.1. In the present study, the respective values for the same age group were 10.9 and 3.0, while in the 2007/2008 study [11], the respective values had been 13.7 and 6.1. This comparison shows that the number of missing teeth is, nonetheless, on average, still distinctly higher in persons with intellectual disability than in persons from the general population.

The current study presents an interesting peculiarity because it is one of the few dental health studies that present data on the prevalence of fissure sealants in adults. It, thus, also allowed analysis of the associations between this figure and the DMFT values. From a scientific point of view, the caries-preventive effect of fissure sealants in children and adolescents has been unquestioned in the last decades [25]. The positive effect of fissure sealants could now also be shown for young adults in our study, aged between 18 and 34 years, as those with at least one fissure sealant showed a statistically significant lower mean D3FMT value than those without fissure sealants (Table 4). Because fissure sealants are usually applied in children and adolescents, this result can be interpreted as a long-term caries-preventive effect. In this context it should be mentioned that, at 50%, the proportion of study participants with at least one fissure sealant was considerable in the age group of 18 to 34 years old, whereas this proportion was only 12.5% in the age group of 35 to 54 years old (Table 2). This age-related difference in the incidence of fissure sealants is very probably due to the fact that the German statutory health insurances, which provide coverage for about 90% of the German population, began reimbursing dentists for this preventive measure in 1993 [26,27]. Since then, German dentists have also been reimbursed for other preventive measures in children and adolescents, such as assessment of oral hygiene, dental health education, and application of fluoride varnish [26]. In addition, around the same time, dentists employed by the regional health authorities were legally tasked with educating school children on caries prevention. These dentists are also entitled to apply fluoride varnish to children’s teeth during dental examinations in schools, on the provision that the children’s parents have given their consent. Initially, the latter two measures were only intended for children aged 6–12 years, but in 1997, they were extended to also include children of other ages with an increased risk for caries (e.g., children with disability) [27]. Another caries-preventive measure that deserves mention is the availability of fluoridated salt in German supermarkets since 1991. In fact, since 2005, the market share of fluoridated salt has lain between 50% and 68% [2,28]. Although fissure sealants are the only preventive measure to be visible in oral epidemiological studies, due to the fact that they usually remain visible for a long time, it is highly likely that the participants in our study benefitted from all of the caries-preventive measures mentioned above. The authors would like to stress this point to avoid the impression that the success of caries prevention in Germany is due solely to the application of fissure sealants. Thus, the epidemiological assessment of caries-preventive measures should avoid taking only the effect of fissure sealants into account. That said, the authors of the present study recommend including the presence of fissure sealants in young adults as a parameter in future regional or national oral epidemiological studies. This practice would provide a sound basis for conclusions about the long-term effect of preventive measures, fissure sealants included, in adults of the general population.

The presented study has some limitations. For one, information in regard to the number of years that each study participant had been occupied in the sheltered workshop was not available. In addition, information in regard to when participants in Groups 2 and 3 stopped living with their parents was also not available. In this study, the study sample comprised 15.6% of the persons with intellectual disability who were occupied in the sheltered workshops in the study region. This percentage is lower than the 21.9% in the study sample in the previous German study carried out ten years before. Despite the high efforts of the officer of the Perthes foundation who was responsible for the coordination of the examination schedule, the integration of dental examinations for the study into the daily processes in the sheltered workshops proved very complicated. In addition, for unknown reasons, some legal guardians are apparently hesitant to allow their wards to participate in studies.

It was also not possible to obtain valid data on the periodontal status of the study participants, mainly due to the limited cooperation of these persons in accepting the use of periodontal probes. In light of the increasing, or, at least, considerable, prevalence of periodontitis in the general population [29,30] and the decreasing number of teeth missing due to caries, it is to be expected that this oral disease will also gain more relevance for persons with intellectual disability. In systematic oral health reviews on persons with disability, an increased prevalence of periodontal diseases has been described [8,9]. In this context, one should keep in mind that the increased prevalence of periodontal diseases is not always due to insufficient oral hygiene. Genetic factors can also be one of the reasons for periodontitis in persons with disability, as has, for example, been described for persons with Down syndrome over 20 years ago [31,32]. The authors of the present study, therefore, also recommend the assessment of periodontal health in future oral epidemiological studies on persons with disability. In addition, it should be noted whether a syndrome was diagnosed in the study participants or not. Experts in periodontal epidemiology are also encouraged to find solutions addressing how best to assess the prevalence of periodontitis in persons with intellectual disability.

## 5. Conclusions

In conclusion, and despite the limitations of this regional study, the results of the current study indicate that the dental health of adults with intellectual disability has improved in Germany in recent years. In addition, the dental health status in this subgroup of the population seems to be approaching that of the general population. Nonetheless, considerable inequality still remains, given that persons with intellectual disability, on average, still have more missing teeth than persons in the general population. Particularly for persons with intellectual disabilities, who frequently display low tolerance for restorative treatment, prevention is of considerable importance. The present study illustrates that preventive measures, such as application of fissure sealants and fluoride varnish in childhood and adolescence, have long-lasting positive effects on the dental health of persons with intellectual disability, which persist into young adulthood. The application of these measures should thus not cease with the end of adolescence, but should be continued during adulthood. Moreover, oral epidemiological studies on this population group should be conducted at regular intervals and cover all regions of a country, not just a few. The design of these future studies should also include information on the living arrangements of the persons with disability included in the investigations to assess potential associations between sociodemographic factors and oral health.

## Figures and Tables

**Table 1:** Age, gender and living arrangements of the study participants, as well as information on the persons who completed the questionnaires.

Persons with Intellectual Disability	Frequencies *n* = 132	
**Gender**		
Male	65	49.2%
Female	67	51.8%
**Age (in years)**		
Mean all ± (SD)	35.2 ± 12.7	
Range	18–69	
Mean male ± (SD)	35.8 ± 13.1	
Range	18–65	
Mean female ± (SD)	34.5 ± 12.3	
Range	18–69	
**Living arrangement**		
Alone	11	8.3%
At parent’s home/at a family member’s home	81	61.4%
In a supervised institution/assisted living facility	28	21.2%
In a shared apartment/with the partner	7	5.3%
Other	4	3.0%
Unstated	1	0.8%
**Characteristics of Persons, who completed the Questionnaire**		
**Relationship of answering person to person with intellectual disability**		
Mother	64	48.5%
Father	11	8.3%
Parents together	1	0.8%
Another familiarly relationship	16	12.1%
Legal guardian, but not in familiarly relationship	5	3.8%
Another person, working in cared living facilities or workshop	31	23.5%
No statement	4	3.0%
Person, who completed the questionnaire, was the legal guardian	80	60.6%

**Table 2:** Prevalence rates for persons with intellectual disability without caries experience, without treatment need, or with treatment need, in different groups.

Groups (Sorted by Living Arrangements)	*n*	Mean Age (in Yrs)	Caries Free, % (D3MFT = 0)	Without Treatment Need, % (D3T = 0 and MFT > 0)	With Treatment Need, % (D3T > 0)	With Fissure Sealant, % (FS >0)	With Prosthodontic Restorations, %
Group 1	81	31.2	8.6	72.8	27.2	38.3	22.2
Group 2	28	42.0	10.7	71.4	28.6	28.6	35.7
Group 3	22	40.4	18.2	77.3	22.7	18.2	50.0
unstated	1	48.0	0	0	100	0	100
All	132	35.2	10.6	72.7	27.3	32.6	30.3
Age groups							
18- to 24-year olds	32	21.5	15.6	53.1	46.9	53.1	6.3
25- to 34-year olds	42	28.7	19.0	85.7	14.3	47.6	14.3
35- to 44-year olds	24	39.1	4.2	75.0	25.0	20.8	37.5
45- to 54-year olds	23	49.8	0	69.6	30.4	4.3	60.9
55- to 69-year olds	11	60.5	0	72.8	18.2	0	81.8

**Table 3:** Mean DT, MT, FT and D3MFT values and standard deviation (SD) according to the participant’s age group, gender and living arrangement.

Groups	Mean Age (in Yrs)	*n*	Mean D3T (±SD)	Mean MT (±SD)	Mean FT (±SD)	Mean D3MFT (±SD)	Mean D3MFT (95% CI)	Caries Prevalence% (D3MFT > 0) 95% CI
All	35.2	132	0.5 (1.1)	4.3 (7.2)	4.7 (4.9)	9.5 (8.6)	9.5 (8.1–11.0)	89.4
Men	35.8	65	0.5 (1.1)	4.7 (7.8)	4.7 (4.9)	9.9 (8.8)	9.9 (7.7–12.0)	87.1 (11.3) 2.2–13.5
Women	34.5	67	0.6 (1.3)	4.0 (6.6)	4.7 (4.9)	9.2 (8.4)	9.2 (7.2–11.2)	91.0 (10.1) 8.0–12.2
Groups (sorted by living arrangements)								
Group 1	31.2	81	0.4 (0.9)	2.9 * (5.0)	4.3 (4.8)	7.6 (7.3)	7.6 * (6.0–9.2)	91.4 * 89.6–93.2 (8.4) 6.7–10.0
Group 2	42.0	28	0.7 (1.5)	5.2 (8.5)	4.9 (4.3)	10.8 (9.5)	10.8 (7.2–14.3)	89.3 * 87.5–91.1 (12.0) 8.4–15.7
Group 3	40.4	22	0.7 (1.7)	8.6 * (10.3)	5.7 (6.0)	15.0 (9.6)	15.0 * (11.0–19.0)	81.8 * 80.0–83.6 (18.3) 15.1–21.6
unstated	48.0	1	n/a	n/a	n/a	n/a	n/a	n/a
Age groups								
18–34 yrs	25.6	74	0.4 (0.8)	1.2 (2.4)	3.3 (4.1)	4.9 (5.1)	4.9 (2.3–6.0)	82.4 (5.9) 4.6–7.2
35–44 yrs	39.4	24	0.4 (1.1)	3.0 (3.5)	7.4 (5.5)	10.9 (6.3)	10.9 (8.4-13.4)	95.8 (11.3) 8.9–13.8
45–69 yrs	53.3	34	0.9 (1.8)	12 (10)	5.9 (5.0)	18.8 (8.3)	18.8 (16.0–21.6)	100 (18.8) 16.0–21.6

* statistically significant.

**Table 4:** Association between fissure sealants and D3MFT-value in 18- to 34- year-old adults.

Group 18–34 Yrs	FS > 0	FS = 0
mean age (in yrs)	25.5	25.6
*n*	37	37
mean D3T (±SD)	0.2 (0.6)	0.6 (0.9)
mean MT (±SD)	0.7 (1.3)	1.6 (3.0)
mean FT (±SD)	2.1 (3.0)	4.5 (4.7)
mean D3MFT (±SD)	3.0 * (3.6)	6.7 * (5.7)
mean D3MFT—95% CI	1.9–4.2	4.9–8.5
caries prevalence% (95% CI) D3MFT > 0 (95% CI)	75.7 (62.4–89.0) 4.0 * (2.7–5.3)	89.3 (76.0–102.6) 7.5 * (5.6–9.4)

* statistically significant.

## Data Availability

The data presented in this study are available on request from the corresponding author. The data are not publicly available due to privacy reasons.
